# Benefit of virtual reality during visceral artery aneurysms open and endovascular surgery planning

**DOI:** 10.1016/j.jvscit.2025.101997

**Published:** 2025-09-24

**Authors:** Delphine Donzel, Dominique Fabre, Thomas Le Houérou, Antoine Gaudin, Alessandro Costanzo, Stephan Haulon

**Affiliations:** Paris-Saclay University, Marie Lannelongue Hospital, Le Plessis-Robinson, France

**Keywords:** Virtual reality, Aneurysm, Treatment selection, Surgical planning

## Abstract

**Objective:**

To assess the influence of three-dimensional (3D) virtual reality (VR) analysis on surgical decision-making in visceral artery aneurysms (VAAs) surgery, aiming to optimize treatment selection and surgical planning.

**Methods:**

A total of 10 patients with VAAs were selected. Ten surgeons were each assigned to analyze three of these aneurysm cases. Contrast-enhanced computed tomography scans were initially analyzed with standard two-dimensional multiplanar reconstructions and subsequently with 3D VR reconstruction using a VR headset running the Avatar Medical Vision software. Surgeons were required to choose between managing the aneurysm via open repair (OR) (resection-anastomosis, aneurysmorrhaphy or bypass) or endovascular procedure (EP) (embolization and/or stenting). After VAA analysis and procedure planning, surgeons completed a qualitative variable-based questionnaire using a discontinuous scale of approval levels. The time taken to analyze VAAs using VR was also recorded.

**Results:**

Ten patients (aged 55-76 years) with VAAs involving four splenic arteries, two renal arteries, one celiac trunk extending to the splenic artery, one patient with both splenic and renal artery aneurisms, and two duodenopancreatic arch aneurysms (one associated with celiac trunk occlusion) were analyzed. Surgeons reported enhanced VAA location, anatomy assessment, and collateral diagnosis (83%, 90%, and 76%, respectively) using VR. Improved procedure planning and anticipation of challenges was also reported (62% and 72%, respectively). The choice of procedure (OR vs EP) was modified in 15% of cases after 3D VR analysis.

**Conclusions:**

This pilot study demonstrates consistent results regarding the use of VR for preoperative evaluation of patients with VAAs. The learning curve is short, and the analysis process is not time consuming. VR is a promising tool to optimize treatment selection (EP or OR).

Visceral artery aneurysms (VAAs) are an uncommon condition typically encountered in patients with either atherosclerotic, genetic, or inflammatory disease. The renal arteries are the most frequently affected site, followed by the splenic, hepatic, pancreaticoduodenal, and gastroduodenal arteries.^1^ VAAs appear to progress slowly, with the absolute risk of complications varying by location.2., 3., 4. Nonetheless, the natural history of VAAs may include rupture or erosion into an adjacent viscera, both of which expose patients to hemorrhage and a high risk of mortality.^5^ Indications for treatment are now well-defined with guidelines outlining the therapeutic strategy for each artery.^2^ When required, surgical exclusion via open repair (OR) or via endovascular procedure (EP) are the primary therapeutic options and both technical approaches have demonstrated favorable outcomes.^6^ A computed tomography (CT) scan is essential for the preoperative analysis of VAAs, but VAA anatomy can be complex and challenging to interpret. Novel visualization tools based on computer-assisted three-dimensional virtual reality (3D VR) CT scan reconstructions have been developed and shown to be beneficial for visualizing vascular anatomy. This study aimed to assess the influence of 3D VR footage analysis for surgical decision-making in VAAs to optimize treatment selection (OR vs EP) and surgical planning.

## Methods

### Patient selection

Ten patients (aged 55-76 years) with VAAs were selected among those who underwent a surgical procedure in our department during a 3-year period from January 1, 2020, to December 31, 2022. The cohort comprised four splenic artery aneurysms, two renal artery aneurysms, one celiac trunk aneurysm that extended to the splenic artery, one patient with both splenic and renal artery aneurysm, and two duodenopancreatic arch aneurysms, of which one was associated with a celiac trunk occlusion. We retrospectively analyzed these cases, which had already undergone OR or EP, to assess the influence of the 3D-VR analysis on surgical technique selection. Cases were analyzed by surgeons blinded to the repair that had been performed, to evaluate the difference between standard two-dimensional (2D) analysis and 3D-VR analysis. The choice between OR and EP, was at the surgeon's discretion after 3D analysis. Open surgical techniques included resection-anastomosis, aneurysmorrhaphy or bypass. EP included embolization or stenting. The analysis focused only on scanner visualization. Surgeons were also blinded to patient's comorbidities. Preoperative contrast-enhanced CT scans had been preoperatively performed as a part of our standard patient workup. All surgeons participating in the analysis were part of the surgical team. The patients consented to having their data processed for scientific research purposes. All clinical cases were anonymized and designated by a number throughout the study. No institutional review board approval was required for this retrospective observational study on anonymized CT scans.

### Image analysis

#### Standard 3D viewer analysis

**Fig 1:**
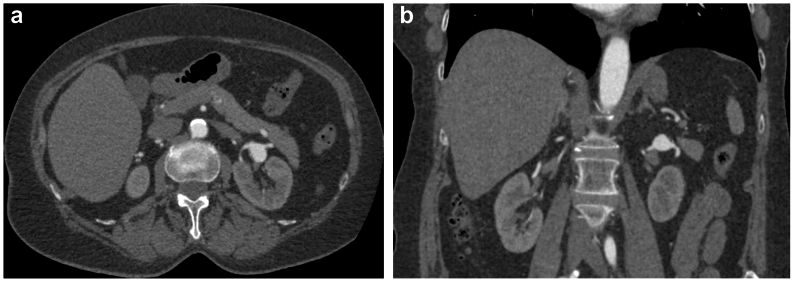
Patient with an aneurysm in the left renal artery in an ordinary mode of two-dimensional visualization with Aquarius iNtuition Viewer (Terarecon). **(A)** Axial section. **(B)** Coronal section.

Digital Imaging and Communications in Medicine (DICOM) files were acquired during routine follow-up. Initial CT scan analysis was conducted using the Aquarius iNtuition Viewer (Terarecon). This software enables navigation in a 2D multiplanar reconstruction (2D MPR) mode of visualization on a conventional computer screen. Although 3D vascular reconstructions can be generated, the software does not support 3D VR visualization (Fig 1). The Aquarius iNtuition Viewer is routinely used in our hospital for image analysis and surgical planning for all patients. Therefore, all interviewed surgeons were familiar with its operation.

#### Three-dimensional VR analysis

**Fig 2:**
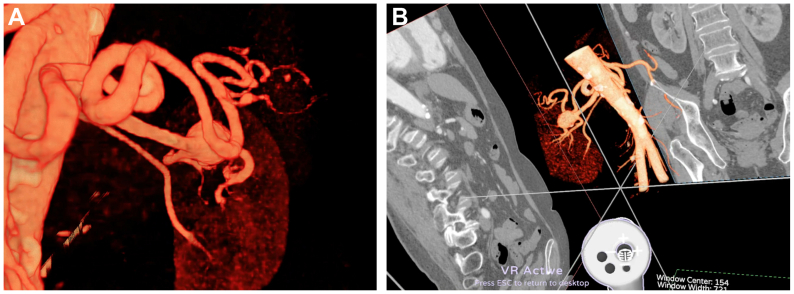
Same patient from Fig 1. with an aneurysm in the left renal artery in a three-dimensional virtual reality (3D VR) mode of visualization after reconstruction by AVATAR MEDICAL 3D VR software. **(A)** A representation close to reality is obtained using the VR headset. **(B)** The cropping tool allows the user to isolate the zone of interest, zooming and rotating the aneurysm permit the appreciation of its anatomy.

**Fig 3:**
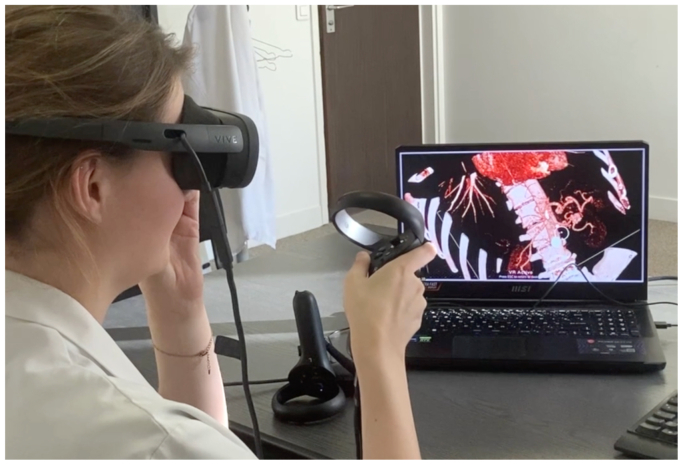
Virtual reality (VR) headset and handheld device are easily used to run AVATAR MEDICAL software.

Three-dimensional VR imaging was obtained after reconstruction using the Avatar Medical Vision software. This software has been developed over recent years and is designed to enhance visualization and surgical planning. It has received clearance (510(K) route) from the US Food and Drug Administration and CE mark class IIa in 2025. Image processing by Avatar Medical Vision software generates 3D VR renderings from any DICOM file. Unlike most VR medical imaging solutions, which rely on a preliminary segmentation step that simplifies the image by transforming it into surfaces, Avatar Medical Vision uses volume rendering of the CT scan and leverages the latest VR game engines to deploy it in a stereoscopic context (Fig 2). A VR headset and a handheld device were used to operate the Avatar Medical Vision software (Fig 3). The handheld device permitted 3D navigation within the patient's vascular anatomy. Viewing filters were optimized by a clinical solution specialist from Avatar Medical in collaboration with two surgeons. Videos of the software in use are available in the appendix (online only). All surgeons underwent specific training lasting approximately 15 minutes before using Avatar Medical Vision and its associated devices. This training enabled the surgeons to acquaint themselves with the various tools offered by the software through a demonstrative clinical case.

### Perception questionnaire

Ten surgeons (five senior vascular surgeons and five vascular surgeons in training) from our vascular surgery department were recruited for the study. Three cases were randomly assigned and analyzed by each surgeon, ensuring that each case was reviewed by at least three surgeons (two seniors surgeons and one surgeon in training, or one senior surgeon and two surgeons in training). Data collection was prospective at each step of image analysis. A data collector was assigned to interview the surgeons and to collect their answers prospectively without the option to revisit previous steps. For each case, a detailed analysis was conducted using a three-part questionnaire (Table). A first part involving surgical indication and planning was submitted to the surgeon after an initial analysis of the standard 2D MPRs with Aquarius iNtuition software (Table). Each surgeon could use the Aquarius iNtuition software's tools according to their usual practice. The surgeon had to indicate whether a surgical intervention was warranted for the case. Subsequently, the surgeon was asked to detail the footage analysis by counting the efferent arteries coming from the aneurysm and to specify his surgical strategy. The final question inquired about the surgeon's choice between OR and EP. The second step involved a 3D VR analysis of the same case using the Avatar Medical Vision software immediately followed by a qualitative variable-based questionnaire (Table). This subjective perception questionnaire aimed to evaluate the benefit of using 3D VR analysis compared with 2D analysis in terms of contribution for image analysis and surgical planning. Responses were scaled with a 5-level agreement scale ranging from “I totally agree” to “I do not agree,” with an intermediate option of “I see no difference.” Surgeons were queried about the ease of use in determining aneurysm location, aneurysm anatomy, identification of collateral branches, surgical planning, and the possibility of anticipating potential surgical challenge. The surgeon's overall impression of the device was thus collected. The third part aimed to assess whether 3D VR analysis could modify surgical decision (Table). Questions from the first questionnaire were repeated in the same order to identify any modification from the 2D analysis compared with the 3D VR analysis. In cases where the surgeon reconsidered the indication for surgical intervention (question 1 from part 3: “Do you retain an indication for surgical intervention?”), the surgeon could access both the Aquarius iNtuition Viewer software and the Avatar Medical Vision software to confirm their decision before providing their definitive answer. Ultimately, surgeons were asked again to choose between OR and EP to identify any major modifications in the overall surgical strategy.

**Table I:** Three-part questionnaire

Part 1: CT scan analysis with standard 2D software
1. Do you retain an indication for surgical intervention? Yes/No 2. How many efferent arteries do you count? 3. How many arteries do you plan to save? 4. How many arteries do you plan to sacrifice? 5. Do you plan an open repair or an endovascular procedure?
Part 2: Subjective qualitative
1. I can locate the aneurysm better with AVATAR MEDICAL compared to Aquarius. I totally agree. I tend to agree. I see no difference. I rather disagree. I do not agree.
2. I can see better the anatomy of the aneurysm with AVATAR MEDICAL compared to Aquarius. I totally agree. I tend to agree. I see no difference. I rather disagree. I do not agree.
3. I have a better idea of the collateral branches and their size with AVATAR MEDICAL compared to Aquarius. I totally agree. I tend to agree. I see no difference. I rather disagree. I do not agree.
4. I better plan my surgery with AVATAR MEDICAL compared to Aquarius. I totally agree. I tend to agree. I see no difference. I rather disagree. I do not agree.
5. I can better anticipate the degree of difficulty of the gesture with AVATAR MEDICAL compared to Aquarius. I totally agree. I tend to agree. I see no difference. I rather disagree. I do not agree.
Part 3: CT scan analysis with 3D VR AVATAR MEDICAL
1. Do you retain an indication for surgical intervention? Yes/No 2. How many efferent arteries do you count? 3. How many arteries do you plan to save? 4. How many arteries do you plan to sacrifice? 5. Do you plan an open repair or an endovascular procedure?

*2D,* Two-dimensional; *3D,* three-dimensional; *CT,* computed tomography; *VR,* virtual reality.

## Results

### Image analysis: Standard viewer analysis vs 3D VR analysis

A total of 30 case analyses were obtained from 10 surgeons. One case analysis was incomplete and was not included, so the final analysis was performed on 29 cases. In seven cases (24.1%), the number of identified efferent branches differed between the 2D and the 3D VR analyses. An additional branch was identified in five cases and one less branch was identified in two cases after 3D VR analysis. The additional branches were those not initially identified during 2D analysis, and the unidentified branch was a branch wrapped around the aneurysm that was identified during the 3D VR analysis. Among the 22 cases with the same number of counted branches in both the 2D and 3D VR analyses, the number of branches to be occluded changed for six cases (27.3%). In these six cases, the decision to occlude fewer branches was made in four cases, and the decision to occlude more branches was made in two cases. After the 3D VR analysis, a different surgical approach was chosen by the surgeon in 4 of the 29 analyzed cases (13.7%). Among these four cases, the surgical approach changed from OR to EP in three cases, and from EP to OR in one case.

### Perception questionnaire results

**Fig 4:**
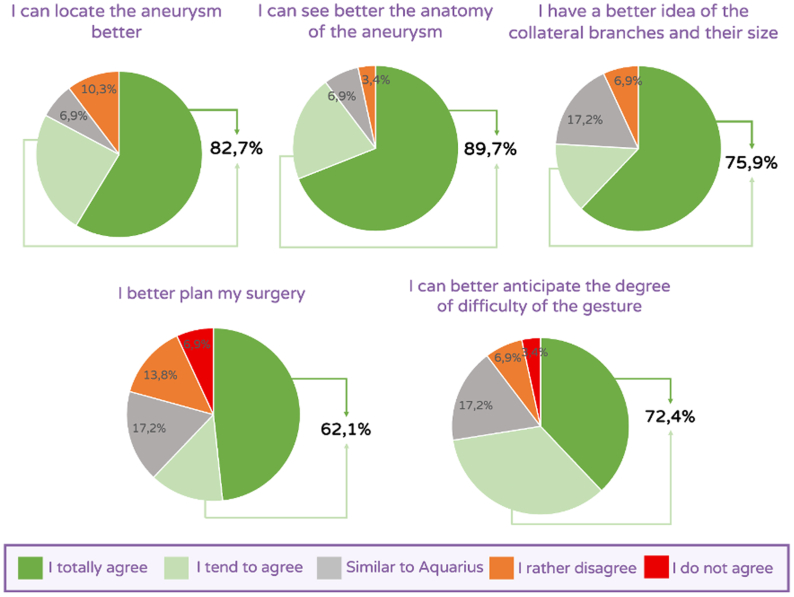
Results from subjective perception questionnaire presented in Table.

The results are presented in Fig 4. The perception questionnaire indicated that a vast majority of surgeons experienced enhanced VAA location and an improved assessment of its anatomy during 3D VR analysis (82.7% and 89.7%, respectively). A better assessment of collateral branches was also reported (75.9%). Most surgeons reported improved surgical procedure planning and easier anticipation of potential surgical difficulties (62.1% and 72.4%, respectively).

## Discussion

Advancements in clinical practice are being driven by innovative technologies that enhance presurgical anatomy visualization across various surgical specialties. Previously, 3D VR devices were time consuming to operate owing to their complexity, but they have since become more user friendly, as evidenced by a reduced learning curve.^7^ In our study, each surgeon proficiently learned to use Avatar Medical Vision in a short period.

The high level of satisfaction expressed by surgeons suggests that 3D VR analysis offers new perspectives in terms of vascular anatomy analysis and presurgical planning. The 3D Viewer enhanced details of the lesion anatomy. The limitation of standard viewers is the loss of important features when converting 2D images into a 3D reconstruction. Filters were calibrated a priori to enhance specific intensity of iodinated contrast agent, to this end. We noticed a high quality of the reconstructions with Avatar Medical Vision, notably when focusing on small vessels, whereas in our experience, these collaterals are not always visualized when performing 3D reconstructions with standard viewers. During our analysis, the number of efferent branches was revised in seven cases after 3D VR analysis. In five of these cases, surgeons attributed this modification to the enhanced appreciation of branches and anatomical details afforded by 3D VR compared with conventional viewers. Moreover, the stereoscopic vision in VR may aid in the perception of reliefs and relative positioning by adding depth to the images. By better understanding the anatomy of the diseased vessel, surgeons concluded that they would be more comfortable when performing the repair (OR vs EP). Although previous investigations into the impact of 3D VR on surgeons' memory have yielded mixed results8., 9., 10., 11.; preliminary anatomical analysis using this technology can enhance surgeon's confidence by identifying specific anatomical variation and giving a déjà-vu effect, as previously shown in VR neurosurgery planning.^12^ Indeed, Stadie et al^12^ mentioned that even experienced surgeons reported a better preoperative understanding. VAAs often feature complex anatomy, involving division branches that can be challenging to analyze on 2D screens, even for experienced surgeons. They are considered as mostly uncommon pathologies with few indications. Thus, precise anatomical analysis and surgical planning are keys to minimizing occlusion of efferent branches.

Although our work focused on perception and mental image analysis, previous research has shown that 3D VR visualization can also impact the quality of manual measurements.^13^ Kamiya et al^13^ compared 2D MPR vs 3D VR measurements of the aortic root and the proximal part of coronary arteries of a swine heart. Using direct measurements on a silicone cast as a gold standard, the authors suggested that 3D VR reconstructions may enhance measurement accuracy, particularly for curved lines. Notably, a difference was observed for the measurements of cardiac cusps, owing to a challenging appreciation on a 2D MPR mode of visualization. Further detailed analysis using 3D VR tools could guide surgeons in selecting the most appropriate procedure tailored to the patient's specificities. As an example, Kim et al^14^ presented a very interesting work using 3D VR analysis before cardiac surgery coupled with computer simulation. Working with engineers permitted the virtually testing of different vascular grafts and sometimes a reimagining of the procedure before patients underwent cardiac surgery, thus helping to determine the best strategy to adopt. In the field of colorectal surgery, a team documented the use of virtual 3D anatomical models during colorectal surgical procedures.^15^ The 3D VR tool demonstrated significant value in identifying anatomical variations that were overlooked during the 2D analysis, thereby facilitating safe and precise dissection. Such devices have also been shown to be useful in neurosurgery,^16^^,^^17^ craniofacial surgery,^18^ and orthopedic surgery.^19^

Other tools creating an immersive 3D VR environment have been developed and could be integrated in clinical practice for physicians and nurses alike, such as devices to assist needle insertion for vascular access.^20^ The 3D VR system has already been extensively applied to learning anatomy, with positive effects reported on student satisfaction.^10^^,^^11^^,^^21^

Our study has several limitations. First, the questionnaire used was not validated before the study; it was designed by one of the authors, an experienced vascular surgeon. Additionally, although specific training was mandatory before using the Avatar Medical Vision software, surgeons were not tested for proficiency, and responses to the qualitative questionnaire were subjective. Furthermore, surgeons were blinded to patient comorbidities, which have a major impact when choosing between OR and EP. We observed that the viewer could influence the choice of surgical technique (OR vs EP), but we cannot conclude that one technique is superior to the other.

## Conclusions

VR appears to be a promising tool for perioperative management for surgeons. This pilot study suggests interesting results regarding the use of VR for the preoperative evaluation of patients with VAAs. This technology has been well-received by surgeons for the analysis of complex cases such as VAAs. Three-dimensional VR analysis could be integrated in clinical practice to better select the most appropriate surgical approach to perform the repair (OR or EP).

## Funding

None.

## Disclosures

The 3D lens and software were installed in our department at no cost by Avatar Medical during the study period.

## Appendix (online only)

Video Video of the Avatar Medical Vision software's screen return in use. Note that the three-dimensional (3D) effect in virtual reality is not experienced on a standard screen.

Video Video of a tracking shot using the Avatar Medical Vision software. The aneurysm in the left renal artery is the same as in Figs 1 and 2.
